# Porous Structures, Surface Modifications, and Smart Technologies for Total Ankle Arthroplasty: A Narrative Review

**DOI:** 10.3390/bioengineering12090955

**Published:** 2025-09-05

**Authors:** Joshua M. Tennyson, Michael O. Sohn, Arun K. Movva, Kishen Mitra, Conor N. O’Neill, Albert T. Anastasio, Samuel B. Adams

**Affiliations:** 1 Feinberg School of Medicine, Northwestern University, Chicago, IL 60611, USA; joshua.tennyson@northwestern.edu (J.M.T.); michael.sohn@northwestern.edu (M.O.S.); arun.movva@northwestern.edu (A.K.M.); 2 Department of Orthopaedic Surgery, Duke University School of Medicine, Durham, NC 27710, USA; kishen.mitra@duke.edu (K.M.); conor.n.oneill@duke.edu (C.N.O.); albert.anastasio@duke.edu (A.T.A.)

**Keywords:** total ankle arthroplasty, osseointegration, surface modification, porous structures, antimicrobial coatings, smart implants, biointegration, wear resistance, nanotechnology, medical device design

## Abstract

Surface engineering and architectural design represent key frontiers in total ankle arthroplasty (TAA) implant development. This narrative review examines biointegration strategies, focusing on porous structures, surface modification techniques, and emerging smart technologies. Optimal porous architectures with 300–600 µm pore sizes facilitate bone ingrowth and osseointegration, while functionally graded structures address regional biomechanical demands. Surface modification encompasses bioactive treatments (such as calcium phosphate coatings), topographical modifications (including micro/nanotexturing), antimicrobial approaches (utilizing metallic ions or antibiotic incorporation), and wear-resistant technologies (such as diamond-like carbon coatings). Multifunctional approaches combine strategies to simultaneously address infection prevention, enhance osseointegration, and improve wear resistance. Emerging technologies include biodegradable scaffolds, biomimetic surface nanotechnology, and intelligent sensor-based monitoring systems. While many innovations remain in the research stage, they demonstrate the potential to establish TAA as a comprehensive alternative to arthrodesis. Successful implant design requires integrated surface engineering tailored to the ankle joint’s demanding biomechanical and biological environment

## 1. Introduction

Total ankle arthroplasty (TAA) has emerged as a viable treatment for end-stage ankle osteoarthritis [1,2]. TAA prioritizes the restoration of patient quality of life after suffering from end-stage ankle arthritis [2,3]. Modern TAA implants draw upon the results of older implant models, and address issues with implant-tissue integration, mechanical failure, and biocompatibility that have historically impeded clinical performance [3,4,5]. Historically, first-generation ‘cemented-type’ TAA implants experienced challenges regarding accurate implant positioning, cement retrieval, spontaneous malleoli fractures, component subluxation, and mechanical failures; 73% of patients reported consistent pain [4]. Second-generation ‘uncemented-type’ implants aimed to improve osseointegration, mechanical durability, and mobility; this was achieved through the implementation of three-component designs that employed titanium alloys and early surface modifications [4]. A follow-up study investigating the impacts of preparation technology and design development on clinical outcomes analyzed loosening and migration, a significant complication in first-generation implants. While this complication was found to be present in 85% of patients in the cemented implant group, the uncemented, three-component design only displayed a 23% rate of loosening and migration [4]. While these developments made TAA a more viable option for ankle osteoarthritis treatment, TAA implants still faced issues in osseointegration due to difficulties in customizing patient-specific implants; emulating individual patients’ ankle joint biomechanics remained a challenge that contemporary TAA implants strive to improve on [4]. Overall, these upgrades reflect a growing emphasis on bioengineering-driven solutions that improve long-term implant performance and patient outcomes. We define bioengineering-driven solutions as technological innovations that synthesize engineering and biological processes to solve biomedical issues, specifically TAA failures; specific strategies for improvement focus on optimizing patient complication profiles.

Modern TAA success has been enhanced by the utilization of cobalt–chromium and titanium alloys, mobile-bearing configurations, and custom synthetic implant designs. These developments have bolstered both the biomechanical properties and patient clinical outcome profiles temporally [1,4,6,7,8]. While TAA has improved overall, these procedures often still lead to significant complication profiles. Compared to other established joint replacements, such as those of the hip and knee, problematic wound healing, periprosthetic osteolysis, and implant loosening issues, persist in patients who undergo TAA operations, marking potential areas of continued improvement [9,10]. Risk factors associated with TAA failures included age less than 65 years old and BMI ≥ 30 kg/m^2^ [11]. The relatively recent development and constant updates of TAA present another challenge in durability and clinical outcome analysis as limited long-term data are available in the literature [5,12,13]. Careful selection and assembly of TAA implant components must be employed due to the human ankle joint facing forces exceeding five-times body weight during activities such as walking and running [14]. Intentional, calculated TAA implant material selections are necessary to accommodate the high mechanical demands and complex movements of the ankle joint. Cobalt–chromium alloys are often used in articulating talar sections for their superior strength and elastic modulus metrics; titanium alloys are used in tibial sections for their superior osseointegration properties; and polyethylene materials are often used in spacers to emulate natural joint movements [8]. Collectively, these challenges emphasize the necessity of bioengineering innovations such as porous scaffolds and surface modification strategies that aim to improve osseointegration, durability, and complication profiles.

The implementation of porous surface structures of varying sizes has been shown to improve bone ingrowth, and thus osseointegration [15,16,17,18,19,20]. Modifying the sizes, porosity, materials, and regional placement can all impact clinical outcomes in bone–implant interactions [15,16,17,18,19,20,21,22,23,24]. Modifying other bioengineering-based properties, including manufacturing and application methods, can provide greater precision in pore creation and texturing, which can modify durability and biocompatibility [25,26,27]. Additionally, bioactive surface treatments such as calcium phosphate-based and polydopamine-based coatings can serve to improve bonding and stability [28,29,30]. Clinical trials with such coatings have demonstrated a survival rate of 97%, an improvement upon the experiment’s 93% survival rate for hydroxyapatite-coated femoral components in similar patients, in addition to a relatively low wear rate [29]. Surface antimicrobial treatments can prevent bacterial colonization and proliferation at the implant location, improving long-term patient outcomes [31,32]. Modern implantation optimizes positioning by bypassing cystic or compromised bone, addressing challenges faced by previous generations of TAA implants; updated clinical practices combined with the aforementioned bioengineering developments facilitates improved patient outcome profiles [33].

This review explores the current and emerging bioengineering strategies for TAA, synthesizing advances in implant technologies while highlighting historical design parameters that can be optimized to further enhance performance to continue to improve patient outcomes. Porous structure integration, pore geometric variation, and surface texture modification are analyzed for their contributions in bone ingrowth and stability. Surface-level modifications that increase osseointegration, reduce complications, and upgrade durability are assessed. Bioactive coatings, antimicrobial technologies, and other related bioengineering innovations are studied for their osseointegration properties. Finally, emerging advancements in nanotechnology, intelligent implant systems, and “smart” materials are considered for their potential to improve patient clinical outcomes. This narrative review provides researchers, engineers, and clinicians an overview of TAA bioengineering principles, and aims to provide an insight into how advancements in TAA can improve quality of life for patients with end-stage ankle arthritis.

## 2. Porous Structures for Osseointegration

Osseointegration of the porous structures within TAA implants plays a key role in attaining stable fixation. These structures aid bone ingrowth, increase mechanical interlocking, and improve long-term stability. These factors are essential for clinical success. Porous structures facilitate fluid transport and mineral deposition, support bone ingrowth and mechanical interlock, and reduce stiffness mismatch between bone and implant to promote load sharing, thereby creating conditions that enhance apatite formation and that ultimately drive long-term osseointegration [21,26].

### 2.1. Design Principles of Porous Structures

#### 2.1.1. Optimal Pore Size Range

Research on orthopedic implants suggests that pore size has a large impact on the biological response and osseointegration. While the reported thresholds vary, studies consistently identify pores in the 300–600 μm range as the most favorable for promoting bone ingrowth [15,16,17,18]. This range optimizes cellular adhesion, migration, and proliferation. It also permits the development of functional vascular networks, which are necessary for sustained osteogenesis. Scaffold pores can be engineered into spherical, columnar, lamellar, cubical, or other complex 3D-printed geometries, with random spherical pores enhancing osteogenic differentiation; columnar and directional pores improving proliferation, alignment, and nutrient transport; and low-permeability spherical pores promoting chondrogenic behavior [34]. Ultimately, optimal pore shape depends on the target tissue and desired cellular outcome. These structural parameters directly influence the activity of specific cell populations underlying osseointegration: bone marrow stromal PaS cells drive osteogenic differentiation and bone deposition, while immune cells including neutrophils, monocytes, and macrophages regulate the surrounding osteoimmune environment [35,36]. Achieving a foreign body reaction equilibrium between these skeletal and immune cells requires careful control of structural design parameters.

Karageorgiou and Kaplan first demonstrated that pores larger than 300 μm promote new bone formation and capillary infiltration [15]. In contrast, smaller pores (less than 300 μm) tend to create hypoxic microenvironments that impede direct osteogenesis [15]. Mukasheva et al. found that smaller pores in the range of 50–100 μm allowed cell attachment in bone tissue engineering, but that larger pores in the range of 200–400 μm led to enhanced nutrient diffusion and angiogenesis [37]. Klenke et al. similarly found that 140 μm was a critical threshold to surpass for functional capillary density [38]. Luo et al. showed that porous tantalum scaffolds with 400–600 μm pores allow superior cell adhesion, proliferation, and bone integration compared to scaffolds with either smaller or larger pores [16]. Similarly, Taniguchi et al. and Ran et al. independently found that titanium implants with pore sizes close to 600 μm resulted in the most rapid and robust bone ingrowth and mechanical fixation [17,18]. More recent preclinical studies have proposed that larger pore sizes (700–1200 μm) may further improve osteogenesis in bioceramic scaffolds by increasing microfluidic flow and shear stress to optimal levels [19,20]. However, these findings are based on animal models and mathematical modeling and have not yet been validated in human clinical studies.

Other groups have explored the effects of nanoporous structures on osseointegration. Kopf et al. found improved protein adsorption and early blood coagulation to promote cell adhesion when exploring nanoporous structures and hydrophilicity [39]. Effects on stem cell adhesion, proliferation, and osteogenic differentiation from nanoporous structures have been found through pathways like Piezo1-Ca^2+^ activation and β-catenin stabilization [40]. Furthermore, nanoporous surfaces modulate the immune response by promoting macrophage polarization to the pro-healing M2 phenotype, reducing oxidative stress, and fostering a regenerative osteoimmune environment [41,42]. Hierarchical nanoporous designs can sustain ion release and angiogenic signaling to support vascularization alongside osteogenesis [43].

The ability to precisely control pore dimensions is a key factor in optimizing the biological response to implant surfaces. These considerations have important implications for the design and long-term performance of TAA implants.

#### 2.1.2. Porosity and Mechanical Properties

The relationship between porosity percentage and mechanical strength is a fundamental consideration in the design of TAA components. Increased porosity typically improves biological fixation with greater surface area and more interconnected pathways for bone ingrowth, but it also decreases mechanical strength and stiffness. Clinicians and engineers must carefully manage this trade-off to ensure both successful osseointegration and structural integrity under physiological loading conditions [21,22]. Common mechanical properties that TAA implants are evaluated on include loading on multiaxial compressive, tensile, shear, and torsion forces; micromotion at the bone–implant interface and stress shielding; contact stresses and pressures; fatigue strength and wear resistance; and range of motion under cyclic loading [44,45,46,47,48].

To handle competing mechanical and biological requirements, researchers have developed modern porous metals with open-cell architectures for orthopedic applications. Wang et al. showed that precise control of pore size, interconnectivity, and overall porosity allows for the tuning of mechanical properties to more closely match those of native bone, minimizing stress shielding while maintaining sufficient load-bearing capacity [25]. However, porosity levels over a range of 80–90% can compromise implant strength and increase the risk of mechanical failure, while porosities under 50% may limit bone ingrowth and impede long-term fixation [22,49].

#### 2.1.3. Regional Design Considerations

Different regions of TAA implants may benefit from customized porous designs that adjust for the distinct mechanical environments and patterns of bone–implant interaction. For example, studies have shown that the central region of the tibial component permits greater bone ingrowth than the anterior region, suggesting that targeted surface modifications could improve fixation in specific areas [23]. Similarly, finite element analyses of talar components reveal variations in stress distribution and micromotion across regions, reinforcing the importance of tailoring pore architecture to local biomechanical demands [50].

Other studies have emphasized the benefits of bimodal porous distributions, finding that combining two distinct pore size ranges can synergistically benefit osteoblast maturation. In contrast, combining more than two pore size ranges in multimodal distributions did not lead to any improvements relative to unimodal distributions [51].

The concept of functionally graded porous structures has gained attention as a strategy for next-generation TAA designs. These designs have porosities that gradually transition to align with regional requirements. Although clinical studies in TAA are still limited, broader orthopedic and biomaterials research indicates that graded porosity can increase both mechanical stability and biological integration. Design parameters for functionally graded porous structures include the choice of unit cell, the optimal porosity distribution laws, and the volume fraction indices with some studies finding power laws, honeycomb unit cells, and careful control of edge distributions as some factors that could optimize implant design [52,53,54]. Recent reviews of TAA implant technologies have elucidated a growing interest in these approaches, especially for managing the complex loading conditions and osseointegration needs of the tibial and talar components [55].

### 2.2. Manufacturing Methods for Porous Surfaces

#### 2.2.1. Sintering Techniques

Sintering is a traditional method for creating porous surfaces on metallic implants. The process involves the partial fusion of metal particles at temperatures below their melting point, resulting in an interconnected pore network with variable architecture. While this technique can produce moderate porosity suitable for osseointegration, it provides limited control over key geometric features, such as pore size, shape, and distribution, relative to more advanced fabrication methods [25,26].

Sintered surfaces typically contain moderate porosity and heterogeneous pore characteristics. These features can satisfy basic fixation requirements but lack the precision needed to match the mechanical properties of the host bone. Modern implant designs increasingly replace sintering with advanced manufacturing techniques to attain fully customizable pore architectures. Emerging approaches, such as 3D printing and laser sintering, enable careful control over porosity parameters to thereby optimize both mechanical performance and biological integration [56,57].

#### 2.2.2. Plasma Spray Applications

Plasma spray technology is a conventional method used to create textured surfaces on orthopedic implants. By projecting partially melted metal or ceramic particles onto the implant surface, a rough coating is formed with moderate porosity that improves surface roughness and promotes initial fixation. However, this process has limitations in promoting long-term osseointegration and the durability of load-bearing implants [26,27].

Due to these limitations, there has been a shift towards advanced manufacturing techniques that allow greater control over surface architecture. Methods such as 3D printing and laser-based melting enable the pinpoint customization of pore size, shape, and distribution, thereby facilitating a more accurate replication of the cancellous bone structure. These innovations improve both mechanical performance and biological integration to overcome the shortcomings of traditional plasma spray approaches [56].

#### 2.2.3. Additive Manufacturing Approaches

Additive manufacturing has dramatically advanced the fabrication of porous structures for orthopedic implants, including components used in TAA. Techniques such as selective laser melting (SLM) and electron beam melting (EBM) allow for the creation of complex porous architectures with minute control over pore size, shape, and interconnectivity [56,58]. This level of architectural precision is essential for optimizing mechanical properties and improving biological performance by promoting osseointegration and aiding vascularization [25,59,60]. Procedures for the additive manufacturing of TAA implants have been introduced, and computational models have validated some of the benefits of these approaches [61,62].

Additive manufacturing encompasses various techniques, each with distinct advantages for TAA applications. Powder bed fusion methods, including selective laser melting (SLM) and electron beam melting (EBM), are particularly relevant for creating complex, load-bearing porous structures with meticulous geometric control. While SLM offers superior surface finish and dimensional accuracy, EBM provides faster build rates and reduced residual stresses. Direct energy deposition techniques enable rapid prototyping and repair applications, though with lower resolution than powder bed methods [56].

Additive manufacturing has enabled several emerging approaches of implant design, such as functionally graded porous structures and triply periodic minimal surfaces (TPMSs). Functionally graded porous structures allow for greater strength in load-bearing areas while maximizing bone ingrowth in areas where biological integration is important, improving integrity and long-term implant stability [63]. The freedom and flexibility of additive manufacturing help it overcome the limitations of conventional methods to allow for the fabrication of complex geometries. TPMS has gathered interest for its ability to create biomimetic surfaces through the careful, mathematical control of geometry to optimize surface area to volume ratios, permeability, and interconnectivity while minimizing stress concentrations. In doing so, cell adhesion, integration, and vascularization are promoted while stress shielding is limited [64].

#### 2.2.4. Surface Texturing Methods

Various surface modification techniques, including laser-based surface texturing, can increase the osseointegration potential of TAA implants. Laser-based techniques enable precise surface modification by generating a variety of microstructures and nanostructures, including grooves, pits, periodic surface patterns, and bionic textures, which can be tailored by adjusting laser parameters and types [65,66]. Laser texturing alters the implant’s topography, chemistry, and wettability to promote osteoblast adhesion, proliferation, and differentiation. A recent in vitro trial found that relative to grinding and sandblasting, laser texturing best facilitates pre-osteoblast maturation as measured by osteogenic differentiation markers and microscopy [67]. These modifications can yield bone-implant contact and interfacial strength comparable to hydroxyapatite coatings and superior to machined or grit-blasted finishes [68,69].

Additionally, laser-induced microscale and nanoscale surface features further stimulate osteogenic cell activity and increase extracellular matrix mineralization. In vivo studies have communicated that these features lead to improved bone integration compared to conventional implant surfaces [70]. Evidence from preclinical and in vitro research in other orthopedic applications reiterates the potential of laser-textured surfaces, whether used alone or in combination with bioactive coatings, to improve long-term fixation and implant stability [69].

### 2.3. Biological Response to Porous Architectures

#### 2.3.1. Cellular Interactions

The geometric configuration of porous structures plays an essential role in influencing cellular behavior at the bone–implant interface. Using 3D micro-CT and histological analyses, Moreira et al. illustrated that variations in pore shape and size directly affect neo-bone regeneration with specific geometries promoting greater bone ingrowth [71]. Similarly, Bobbert and Zadpoor showed that the orientation of scaffold fibers and struts affects osteoblast adhesion, proliferation, and differentiation, demonstrating the importance of carefully tailored pore designs [72].

Interconnectivity between pores is equally important as it facilitates cell migration, nutrient transport, oxygen transport, and vascularization within the scaffold. Otsuki et al. found that narrow pore throats, especially those with limited pathways to the implant surface, impede tissue ingrowth in porous titanium implants [73]. This finding emphasizes the importance of considering the three-dimensional network structure in addition to the average pore size. Supporting this finding, Lu et al. reported that interconnected bioceramics promote cellular infiltration and bone recolonization in both in vitro and in vivo settings [74]. More recently, Takaoka et al. confirmed that vascularized fibrogenesis precedes bone formation in scaffolds engineered with optimized pore networks in animal models [75]. Motaharina et al. studied the novel implementation of magnesium-based metallic scaffolds in orthopedic implants and their superior biomechanical and osseointegrative capacities, offering yet another promising avenue for TAA upgrades [24]. Together, these studies reinforce the importance of designing porous implants with geometric and interconnectivity features that promote biological transport while maintaining mechanical stability.

#### 2.3.2. Bone Formation Patterns

The process of bone ingrowth into porous implant structures begins with cellular infiltration, follows with progenitor cell differentiation, and concludes with the formation of mineralized bone tissue. Takaoka et al. shared that fibrogenesis and neovascularization occur before mineralization, showing the importance of early vascularization in establishing a microenvironment conducive to sustained osteogenesis [75]. Similarly, Jones et al. used in vivo micro-CT to emphasize the spatial progression of bone ingrowth as typically beginning at the implant periphery and advancing inwards [76]. Synthesizing these concepts, Wu et al. proposed and applied a unified four-stage model of osseointegration (protein adsorption, inflammatory cell adhesion and response, adhesion of additional relevant cells, and angiogenesis and osteogenesis), although they noted that angiogenesis spans all four stages [77]. Optimizing design parameters will allow for the careful tailoring and manipulation of all four stages, increasing their efficiency and effectiveness to shorten healing times and improve outcomes.

The rate and extent of bone ingrowth are controlled by architectural parameters, including pore size, interconnectivity, and throat dimensions, as well as material properties and patient-specific biological factors. Moreira et al. showed that optimized pore geometries improve vascular and cellular infiltration to thereby increase bone regeneration [71]. In addition, Liu and Niebur found that micromotion at the bone–implant interface plays a key role in tissue differentiation [78]. Small displacements allow bone ingrowth and implant stability, whereas larger micromotion promotes fibrous tissue formation and compromises osseointegration. Collectively, these findings provide valuable guidance for designing porous structures in TAA to encourage consistent and durable osseointegration.

#### 2.3.3. Vascularization Factors

Vascularization is a key factor in allowing successful osseointegration as sustained bone formation relies on a sufficient blood supply. Consequently, the design of porous structures must aid not only bone ingrowth, but also the development of a functional vascular network. Xia and Luo emphasized that scaffold architectural cues directly affect neovessel infiltration into pores, which is essential for delivering nutrients and removing waste during bone regeneration [79]. In support of this, Takaoka et al. confirmed in vivo that fibrogenesis and vascularization occur before mineralization to help establish the microenvironment needed for robust bone formation [75].

Architectural parameters, particularly pore size and interconnectivity, play a crucial role in determining vascularization potential. Bai et al. stated that while larger pore sizes increase blood vessel diameter, the size of the interconnections has a greater effect on both vessel density and diameter [80]. Their findings also showed that vascularization tends to plateau at pore sizes greater than 400 μm. Nambiar et al. reviewed a range of strategies to improve scaffold vascularization, emphasizing the use of biological cues, structural modifications, and biophysical stimuli to encourage the formation of dense neovessel networks and to promote a regenerative microenvironment [81]. Optimizing these design parameters is a crucial consideration for porous TAA implants as it can impact both early-stage healing and long-term biological performance.

#### 2.3.4. Integration with Bone

The goal of incorporating porous structures into TAA implants is to accomplish seamless integration with the surrounding bone, resulting in stable biological fixation that resists micromotion and prevents aseptic loosening. Zhang et al. and Quevedo González et al. demonstrated that both implant fixation design and loading conditions affect micromotion at the bone–implant interface [82,83]. Lower levels of micromotion were associated with improved stress distribution, increased implant performance, and greater potential for stable osseointegration.

Modern porous metals, including titanium and tantalum, feature open-cell architectures and mechanical properties that approximate those of native bone. These characteristics promote bone ingrowth and mechanical interlocking at the interface, increasing overall stability [84]. The design of porous structures for TAA implants continues to advance, with emerging fabrication methods enabling the creation of functionally graded architectures tailored to specific mechanical and biological requirements. Li et al. shared hybrid additive manufacturing strategies that combine structural strength with improved bioactivity, while Wang et al. emphasized the value of topological optimization in balancing load-bearing performance with osseointegration [25,85]. Although porous coatings have been shown to improve initial fixation, clinical studies also report potential complications. For example, Togher et al. observed periprosthetic cyst formation in some cases, although this did not appear to increase reoperation rates [86]. Overall, while the incorporation of porous structures in general and the selection of optimized geometrical properties such as larger sizes and higher porosity percentages facilitate enhanced vascularization and osseointegration, mechanical properties such as reduced strength and decreased elastic modulus can result due to interruptions in the constructed implants. Manufacturers and physicians must consider these effects when considering porous structure implementation.

### 2.4. Overall TAA Implant Characteristics

#### Key Determinants of Final TAA Implant Characteristics

The final characteristics of TAA implants are determined by several interrelated factors, most prominently pore size, shape, and interconnectivity. Together, these features regulate cellular behavior, vascularization, and bone ingrowth at the implant interface. Biological responses are strongly influenced by geometry with microscale pores optimizing osteogenesis and vascularization, while nanoporous and hierarchical structures can enhance protein adsorption, immune modulation, and long-term angiogenesis. Porosity percentage also plays a decisive role, as higher porosity increases bone fixation but reduces mechanical strength, creating a necessary trade-off between osseointegration and implant stability. Regional variation in loading conditions further complicates these decisions, as different implant zones benefit from tailored pore architectures or functionally graded porosity to meet site-specific mechanical and biological demands. Manufacturing approaches, including additive techniques and laser texturing, provide the precision needed to fine-tune these parameters and allow customization for both integration and durability. Ultimately, the clinical context dictates how these factors should be prioritized. In some cases, the primary goal may be maximizing vascularization to achieve rapid fixation, short recovery times, and early weight-bearing abilities. In other cases, the focus may be on ensuring sufficient mechanical robustness to withstand high load-bearing demands, such as in younger or more active individuals. In situations where inflammation or infection risk is elevated, as in patients with past periprosthetic infections, modulating immune responses to reduce complications may take precedence. These examples highlight that implant design requires context-specific optimization rather than a universal approach.

## 3. Surface Modification Strategies

The surface characteristics of TAA implants govern their biological integration, mechanical performance, and longevity. Surface modification strategies have evolved to address the complex demands of the ankle joint environment, focusing on increasing osseointegration, preventing infection, and improving wear resistance (Table 1).

### 3.1. Bioactive Surface Treatments

#### 3.1.1. Calcium Phosphate and Hydroxyapatite Coatings

Calcium phosphate-based coatings, particularly hydroxyapatite (HA), are among the most common strategies for improving the biological fixation of orthopedic implants. Their high levels of biocompatibility, osteoconduction, and osseointegration have made them good synthetic bone substitutes in both dentistry and orthopedic surgery [87]. These coatings closely resemble the mineral phase of bone, promoting osseointegration by improving cellular adhesion, proliferation, and osteoblastic differentiation. When applied to TAA components, HA coatings create a bioactive interface that aids direct bone-implant bonding and contributes to early mechanical stability [28,29].

**Figure 1:**
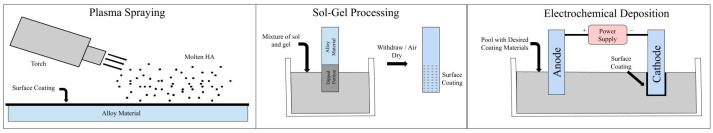
Illustrations of three different methods for applying calcium phosphate coatings: plasma spraying, sol–gel processing, and electrochemical deposition.

Calcium phosphate coatings can be deposited using various techniques, including plasma spraying, sol–gel processing, and biomimetic methods [88,89]. Plasma spraying remains the most widely used clinical approach despite its limitations related to coating uniformity and control over crystalline phases. In contrast, alternative methods, such as sol–gel, in which implants are dipped into a mixture of sol and gel prior to being withdrawn and air dried, and electrochemical deposition, in which a power supply is used to coat the surface of the cathode with a desired material, permit better control of microstructure and composition [88,90]. The schematics of these coating processes are visible in Figure 1. However, these approaches are less common in clinical settings due to challenges in scalability and mechanical durability, especially in high-load applications.

A major challenge for HA coatings in load-bearing implants, such as those used in total artificial joints (TAAs), is arriving at an optimal balance between dissolution rate and mechanical integrity under physiological stresses. Ongoing research aims to refine coating crystallinity, improve adhesion to the implant surface, and incorporate functional dopants to increase both early biological activity and long-term mechanical performance [90,91]. Polydopamine coatings provide a potential alternative to HA coatings for manufacturers striving to improve bioactivity; Park and Jung’s study suggests that this coating improves osseointegration while preserving suitable biomechanical properties, opening another avenue for TAA implant improvement [30].

#### 3.1.2. Bioactive Glass Applications

Bioactive glasses are another class of surface treatments that improve osseointegration through controlled surface reactions. Bioactive glasses most commonly contain silicon dioxide (SiO_2_), sodium oxide (Na_2_O), calcium oxide (CaO), and phosphorous pentoxide (P_2_O_5_) [92]. When exposed to physiological fluids, these materials form a biologically active hydroxycarbonate apatite layer that facilitates bone bonding [92]. The combination of ion release and apatite crystallization on the surface promotes osteogenesis. Their chemical composition can be adjusted to tailor dissolution rates and biological responses for specific clinical applications [93].

Incorporating bioactive glass particles into surface coatings has several potential benefits for TAA implants, including accelerated bone formation and early fixation [94]. Bioactive glasses have also exhibited antimicrobial and angiogenic properties [92,95,96]. Similar to calcium phosphate-based coating, these treatments are useful for cementless fixation at non-articulating surfaces at which bone ingrowth is the main driver of long-term fixation. Comparative in vivo studies of 45S5 bioactive glass versus hydroxyapatite have shown several potential benefits of bioactive glasses. In rabbit models, 45S5 bioactive glass had faster rates of bone formation and complete degradation to allow for bone regeneration, whereas hydroxyapatite particles did not fully degrade [92].

Despite their benefits, bioactive glasses are inherently brittle. Under the cyclic loading conditions and high loads typical of the ankle joint, this brittleness presents a major limitation. Composite or hybrid coating strategies are often required to improve toughness and fatigue resistance to ensure more reliable long-term performance [97].

### 3.2. Surface Topography Modification

#### 3.2.1. Micro- and Nanotexturing Approaches

Surface topography plays an important role in shaping the biological response to implant materials by influencing protein adsorption, cellular attachment, and tissue integration. Advances in manufacturing and surface processing technologies have enabled the precise control of surface features at both the microscale and nanoscale [98,99]. These modifications are useful on all implant surfaces, but particularly for the tibial and talar interfaces of the implant to maximize interlocking. Microscale and nanoscale topographies have shown effects on cell adhesion, protein adsorption and production, migration, proliferation, differentiation, and gene expression [100]. Some nanostructured surfaces have also shown promise in antifouling as bactericides or as preventers of biofilm formation by impeding bacterial adhesion [101].

Microtextured surfaces improve mechanical interlocking and increase the surface area available for cellular interaction, but the research suggests that hierarchical structures combining both microscale and nanoscale features present synergistic advantages. These benefits include improved bone–implant contact, increased osteoblast differentiation, improved local factor production, and faster osseointegration when compared to surfaces with only one type of feature [98,102,103]. These improvements result not only from direct effects on cell behavior, but also from the modulation of the local immune environment. Specifically, these surfaces can promote M2 macrophage polarization and decrease the expression of proinflammatory cytokines, creating a more favorable setting for bone regeneration [104,105].

#### 3.2.2. Surface Roughness Treatments

Techniques such as sandblasting, grit-blasting, and acid-etching are foundational methods for introducing controlled surface roughness on TAA components. Sandblasting with aluminum oxide or other abrasive particles creates microscale roughness through mechanical abrasion while acid-etching with hydrochloric, sulfuric, or hydrofluoric acid solutions generates characteristic surface textures through selective chemical dissolution. When used sequentially in a process referred to as SLA, grit-blasting and acid-etching produce complex, multi-scale surface features that improve mechanical interlocking and promote osteoblastic activity. This combination has had favorable outcomes in orthopedic implants by accelerating osseointegration and contributing to long-term implant stability [106,107,108].

In addition to improving biological integration, these surface treatments can induce compressive residual stresses in the implant to potentially improve fatigue resistance and overall mechanical performance under cyclic loading. To achieve optimal results, important parameters, such as abrasive particle size, impact velocity, and acid concentration, must be carefully controlled to tailor the resulting surface morphology and to balance both biological and mechanical requirements [106].

### 3.3. Antimicrobial Surface Strategies

Periprosthetic infection is a serious complication in TAA, often requiring implant removal and complex revision procedures. To handle this risk, researchers are developing surface modifications with antimicrobial properties to prevent bacterial adhesion and inhibit bacterial growth at the implant–bone interface [31,32,109]. These approaches include antifouling surface topographies, the incorporation of bactericidal agents such as silver or antibiotics, and smart drug-releasing coatings designed to disrupt biofilm formation [31,110]. Both preclinical and clinical studies, particularly those involving silver-coated and antibiotic-coated implants, have shown reduced infection rates compared to uncoated devices. With regards to silver, a recent in vitro trial found that bacterial growth was reduced in implants with silver contents ranging from 7% to 18%, with *Staphylococcus epidermidis* being especially susceptible to the presence of silver ions [111]. Additionally, a three-layer coating consisting of an inner and outer vancomycin-loading layer in addition to a middle layer consisting of IL-12 containing liposomes embedded in sodium alginate has demonstrated antimicrobial properties in both in vitro and in vivo settings [112]. However, questions remain regarding the long-term efficacy of these coatings and the identification of optimal formulations for sustained antimicrobial performance [32,113].

#### 3.3.1. Metallic and Metal Oxide Antimicrobial Surfaces

Several metallic elements and their oxides possess inherent antimicrobial properties that can be utilized in implant surface design. Silver is especially favored for its broad-spectrum antimicrobial activity and relatively low cytotoxicity when used at controlled concentrations [111,114]. Silver nanoparticles or ions can be incorporated into surface coatings to provide sustained antimicrobial release, which inhibits bacterial adhesion and biofilm formation. These coatings do not impair osteoblast function or impede osseointegration when properly constructed [114,115].

Copper and zinc oxide also display strong antimicrobial effects through mechanisms such as membrane disruption and metabolic interference. These elements can be integrated into surface coatings or doped into calcium phosphate matrices to combine infection control with bone-regenerative potential. Both zinc and copper have been shown to promote osteogenic differentiation and angiogenesis while reducing bacterial colonization, thereby aiding bone cell proliferation in parallel with antimicrobial action [116,117]. These bioactive metals are employed in various forms, including ions and nanoparticles, with release kinetics and concentrations carefully adjusted to maximize antimicrobial efficacy while minimizing cytotoxic effects [114,118].

#### 3.3.2. Antibiotic-Incorporated Surfaces and Novel Approaches

Local antibiotic delivery through modified implant surfaces is a common strategy for preventing infections in orthopedic applications. Techniques such as embedding antibiotics into calcium phosphate carriers or polymer films have been developed to reach high local antibiotic concentrations while minimizing systemic exposure and toxicity [119,120]. These antibiotic-loaded coatings provide sustained and controlled release at the implant site, improving both prophylaxis and treatment of periprosthetic infections. These applications may be most useful in the context of high-risk patients or revision surgeries.

Researchers are also actively exploring novel antimicrobial surface designs that do not depend on antibiotic release. Anti-adhesive modifications, including superhydrophobic textures, nanopatterned topographies, and chemically modified coatings, physically disrupt bacterial attachment and biofilm development. These approaches may decrease the risk of antimicrobial resistance and provide long-term efficacy [31,121]. Additionally, photocatalytic coatings responsive to visible light have emerged as a potential non-antibiotic strategy for controlling implant-associated infections. These coatings generate reactive oxygen species on demand to enable targeted antimicrobial effects against a wide range of pathogens [122].

### 3.4. Wear-Resistant Surface Technologies

Wear-induced osteolysis remains a major concern in arthroplasty as particulate debris generated from implant surfaces can trigger a local inflammatory response. This reaction often results in periprosthetic bone loss, aseptic loosening, and finally implant failure. To handle this challenge, surface modification techniques that improve wear resistance have been developed to minimize particle generation and extend the functional lifespan of orthopedic implants [102,123,124]. Wear resistance is particularly beneficial at articulating surfaces and high-stress contact areas of implants.

#### 3.4.1. Surface Hardening Treatments

Surface hardening treatments such as nitrogen ion implantation and plasma nitriding are used to modify the surface composition and microstructure of titanium alloys. These techniques increase surface hardness and improve tribological performance without altering the bulk mechanical properties of the implant. Nitrogen ion implantation creates a hardened surface layer that boosts wear resistance and helps decrease wear rates under the cyclic loading conditions common in TAA [124].

Similarly, plasma nitriding produces a diffusion-hardened layer enriched with hard ceramic phases, such as titanium nitride (TiN) and titanium di-nitride (Ti_2_N). This treatment improves microhardness, microscratch resistance, wear and erosion resistance, and corrosion and tribocorrosion resistance. It also increases coating adhesion and lowers friction coefficients to make it a favorable surface modification for Ti6Al4V-based implants [125]. Notably, both nitrogen ion implantation and plasma nitriding have been shown to preserve the biocompatibility of the treated surfaces, which is essential for long-term clinical success.

#### 3.4.2. Hard Coatings

Hard coating technologies, such as diamond-like carbon (DLC) and ceramic conversion surfaces, can further improve wear resistance in TAA components. DLC coatings are renowned for their exceptional hardness, low friction coefficients, and outstanding corrosion resistance. These properties decrease wear and minimize the release of metal ions at articulating surfaces. The tribological performance of DLC can be finely tuned by modulating the carbon phase ratio (sp^3^/sp^2^), incorporating dopants, and modifying deposition parameters [126].

Ceramic conversion treatments, including thermal oxidation and plasma-based processes, provide alternative hard surface layers that display strong bonding to the substrate and distinct microstructural features [127]. Despite these benefits, the reliable adhesion of hard coatings to metallic substrates remains a challenge, particularly under the high shear and cyclic loading conditions present in the ankle joint.

Delamination and fatigue failure at the coating–substrate interface have been reported, prompting the development of adhesion-promoting interlayers, such as tantalum and zirconium. Researchers are exploring these advanced multilayer and graded-architecture designs to improve interface stability [128,129]. Continued progress in interface engineering and deposition technologies is essential to ensure the long-term clinical success of hard coatings in TAA implants.

**Table 1:** Surface modification strategies for total ankle arthroplasty implants.

Modification Strategy Category	Primary Purpose	Key Benefits	Main Limitations	TAA Applications
Bioactive Surface Treatments	Enhance osseointegration	Calcium phosphate/HA coatings: bioactive interface, direct bone bonding	Coating durability under high loads	Non-articulating surfaces
		Bioactive glass: controlled surface reactions [28,92]	Bioactive glass brittleness under cyclic loading [90]	Cementless fixation surfaces
Surface Topography Modification	Improve mechanical interlocking	Micro/nanotexturing: increased surface area, enhanced cellular interactions	Parameter control for optimal morphology	All implant surfaces
		Surface roughness treatments: accelerated osseointegration [130]	Manufacturing complexity and standardization challenges	Particularly effective for tibial/talar interfaces
Antimicrobial Surface Strategies	Prevent infection	Metallic antimicrobials (Ag, Cu, Zn): broad-spectrum activity	Balancing antimicrobial efficacy with biocompatibility	High-risk patients
		Antibiotic incorporation: sustained local delivery	Long-term effectiveness	Revision cases
		Novel approaches: anti-adhesive surfaces [111,122]	Resistance concerns with antibiotics	Immunocompromised patients
Wear-Resistant Surface Technologies	Minimize particle generation	Surface hardening: enhanced hardness without bulk property changes	Limited modification depth	Articulating surfaces
		Hard coatings (DLCs): extreme hardness, low friction [123,126]	Coating adhesion challenges under high shear	High-stress contact areas

Surface modification strategies are an important component of TAA design and development with important implications for biological integration, infection prevention, and long-term mechanical performance. Surface engineering approaches, including topographical modifications, chemical treatments, and multifunctional coatings, directly impact implant–bone integration, decrease bacterial adhesion, and improve wear resistance [113,130,131].

Combining multiple complementary surface technologies has shown potential to be the future of TAA implants. Approaches such as integrating bioactive and antimicrobial coatings or mixing hierarchical surface structures with wear-resistant treatments are being explored to satisfy the complex mechanical and biological demands of the ankle joint. These strategies aim to improve implant longevity and clinical outcomes by managing multiple challenges simultaneously [113,130,131].

### 3.5. Choice of Fabrication Method

#### Merits of Each Fabrication Method Based on Desired Implant Characteristics

In choosing which fabrication method might be used for surface modifications of a TAA system, one must consider the device’s functional requirements. If the maximization of osseointegration and biological fixation is the goal, bioactive surface treatments, namely calcium phosphate- and hydroxyapatite-based coatings, are the most widely validated options, though brittleness and limited durability may present as limitations. This is mostly useful in non-articulating surfaces and cementless fixation surfaces, in which durability is less of a concern than fixation. Should mechanical interlocking be the primary goal, such as in tibial/talar interfaces, surface topography modifications such as micro/nanotexturing and surface roughness treatments might be used to enhance cellular interactions. However, tight control of manufacturing parameters is required in this process to avoid the induction of residual stresses. In high-risk patients for which infection is the greatest concern, antimicrobial surface strategies might be used to limit bacterial growth. This must be balanced with the risk for antibiotic resistance and decreased biocompatibility. Finally, in high-stress contact areas where wear resistance is key, surface hardening and hard coatings might be used, though their depth of modification is limited. Ultimately, no single method is optimal in all cases; instead, manufacturing techniques ought to be chosen based on the desired characteristics of the implant. Hybrid approaches that integrate complementary strategies are likely to offer the most promise for the next generation of TAA systems.

## 4. Future Directions

The continued progress in materials science and surface nanotechnology presents a promising new horizon for the development of TAA systems, which may aid in improving clinical outcomes and mitigating existing complications. This section further explores active areas of research that hold promise for improving the next generation of TAA systems (Table 2).

### 4.1. Surface Nanotechnology

#### Biomimetic and Multifunctional Surfaces

The advancement of biomimetic surface technologies holds the potential to mirror the organization and biochemical properties of natural human tissues, allowing for an improved physiologic response to TAA systems. For example, the application of precisely controlled nanoporous coatings resembling host bone has been shown to improve osseointegration, and as such, their utilization holds the potential to speed up fixation times [132,133]. Specifically, a recent in vivo experiment demonstrated that such micro/nano-scale (MNS) biomimetic coatings facilitated enhanced osteogenic differentiation of bone marrow stromal cells, and such a study suggests that clinical application in TAA systems is both feasible and necessary [132]. When these topographical modifications are coupled with the addition of antimicrobial peptides to control for infection and synthetic matrix metalloproteinases to facilitate bone remodeling, they are deemed multifactorial and allow for the mitigation of the two most common causes of orthopedic implant failure—infection and aseptic loosening [134,135]. The development of these surfaces is an active area of investigation with the potential to dramatically improve clinical outcomes, though more ankle-specific literature is necessary to elucidate potential benefits.

### 4.2. Intelligent Implant Systems

#### 4.2.1. Sensor-Based Monitoring Technology

**Figure 2:**
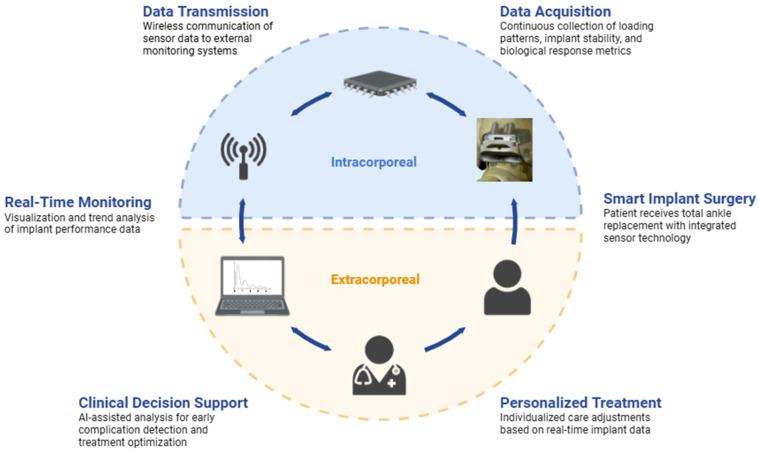
Clinical application framework for intelligent TAA systems. Sensor-enabled TAA implants are integrated with real-time monitoring and decision support to enable personalized patient care.

The integration of sensor technology into TAA implant design is an active area of investigation with the potential to improve clinical outcomes. Miniature sensors within implant components hold the capacity to provide real-time data on loading, bone ingrowth, infection, and implant position, metrics that have traditionally been difficult to measure accurately [136,137,138]. As it stands, load and position sensors can be integrated into implant components to provide real-time data on force transmission, joint loading, joint kinematics, and implant alignment to assess implant function and potential complications [138,139,140]. Similar sensors are already being used in powered ankle exoskeletons (PAEs), in which microprocessors allow for the transmission of information between the human body and the device so as to facilitate a reduction in energy expenditure based on current activity [55]. Emerging biological sensors can detect local temperature changes and pH shifts as an early indicator of inflammation and infection [138,139]. Cosurface capacitive sensing networks have also shown microscale and macroscale monitoring of bone–implant fixation [141]. As illustrated in Figure 2, the data flow from intracorporeal sensors through wireless transmission to extracorporeal real-time monitoring, enabling clinical decision support and individualized care. The increased data that these sensors would provide might allow for the earlier detection of post-operative complications, even before the onset of clinical symptoms, thereby allowing a faster speed of intervention.

Recent improvements in sensor powering have made it increasingly possible to incorporate these sensors into existing implant designs. Energy harvesting systems have demonstrated the ability to convert mechanical and thermal energy in the implant environment into electrical energy; however, whether this amount of energy is sufficient for powering the sensor is a question that warrants further investigation [138]. Additionally, new systems for short-distance, wireless data transmission have shown efficacy, potentially allowing for the increased ease of utilization of these data by patients and providers alike, though wireless data transmission remains an area of concern [142]. Compared to traditional X-ray imaging, these sensor-based methods can potentially improve both cost and time efficiency [143]. Additionally, with recent advancements in the field of artificial intelligence (AI), it must be noted that machine learning might allow for an enhanced assessment of these real-time data, especially once large datasets of patient anatomy and biomechanics are compiled based on sensor data [144,145]. Sensor technologies are already being explored across multiple fields, including arthroplasty, trauma, spine surgery, pediatric orthopedics, infection monitoring, rehabilitation, and sports medicine [146]. As such, the integration of this technology into TAA systems might allow for interventions which are increasingly decided on a patient-by-patient level based on real-time data, facilitating the improvement of clinical outcomes [132,133].

#### 4.2.2. Adaptive Materials

‘Smart’ materials with the capacity to not only sense their surrounding environmental stimuli but also respond to them are another promising avenue regarding future TAA systems. These materials possess the ability to change their properties in response to changes in surrounding variables, such as temperature, pH, and loading, allowing for the improvement of implant performance without requiring intervention from the provider [147,148,149]. Additionally, such materials would facilitate the implant’s response to the dynamic conditions it will encounter at the ankle joint, allowing for enhanced physiological performance. Notably, these materials are not without potential complications. Power management for sensor-equipped implants remains an ongoing area of concern, with energy harvesting systems that rely on mechanical and thermal energy serving as a potential solution [138]. The durability of such materials remains a key consideration. For example, sensor drift, being the movement of sensors that is to be expected following continuous use, is a complication which might be reduced via a sensory array [138]. Additionally, flexibility is a valuable material property so as to ensure durability, with a smart polymer foil coating having recently demonstrated in vitro durability on account of its flexibility [150]. Finally, these materials ought to be biocompatible. While electric components may pose risk of inflammation and toxicity, the utilization of previously discussed surface engineering strategies might help to maximize osseointegration in these implants.

Shape memory alloys (SMAs), although not yet explored in relation to ankle implants, are materials that have demonstrated the ability to shift their configuration in response to mechanical loading. This feature would allow TAA systems to dynamically change shape in response to altered weight-bearing [151]. Additionally, while piezoelectric systems allow for the transmission of mechanical deformations into electrical energy, reverse piezoelectric systems enable mechanical deformation in response to voltage signals, both of which could prove relevant in the dynamic ankle environment [138].

Although these adaptive technologies are still in the research stage regarding orthopedic applications and have not been extensively explored in the context of TAA, they hold the potential to drastically improve the responsiveness and independence of these systems with continued research.

**Table 2:** Future bioengineering directions for total ankle arthroplasty implants.

Future Bioengineering Direction	Primary Purpose	Key Benefits	Main Limitations
Biomimetic/Multifunctional Surface	Enhance osseointegration	Nanoporous coatings mimic bone; improve fixation [132,133]	Limited ankle-specific literature
		Promote osteogenic differentiation of bone marrow stromal cells [132]	Durability of coatings under high cyclic loads is unknown
Sensor-Based Monitoring Technology	Collection and utilization of real-time data on position, loading, and infection	Mechanical information (i.e., loading, position, etc.) can be collected to evaluate function [138,139,140]	Power management of sensor limits lifespan [138]
		Biological markers (e.g., pH, temperature, etc.) serve as early infection markers [138,139]	Wireless data transmission necessary for real-time utilization of data [142]
‘Smart’/Adaptive Materials	Improve implant adaptability to real-time conditions	Dynamic changes in implant properties allow for optimized mechanical performance [147,148,149,151]	Power management, though harvesting may help
		No need for surgeon intervention	Durability of the material, specifically with sensor drift [138]
			Biocompatibility if there are additional electrical components

### 4.3. Clinical Applications

#### Summary of Future Clinical Directions

The next generation of TAA systems will involve increasingly sophisticated approaches that seek to capitalize on past successes while expanding to address challenges that have previously not been efficiently managed. Advances in biomimetic and multifunctional surface nanocoatings allow for faster osseointegration and infection control, while embedded sensor networks enable real-time monitoring for personalized patient care. This will be accomplished through developments in the fields of surface technology and intelligent implant design. Although the majority of these technologies are currently in developmental stages, they represent exciting future directions for the world of TAA implant development. Additionally, the integration of these technologies with existing advances in surgical and rehabilitation techniques holds the potential to expand the value of TAA as a treatment for end-stage ankle arthritis. As such, it is essential to continue monitoring the progress of these developments as the hurdles are overcome.

## 5. Conclusions

Total ankle arthroplasty continues to evolve through advances in porous structure implementation, surface engineering, and osseointegration techniques. Porous structures with tailored dimensions, porosity levels, and regional placement variation foster improved bone ingrowth and mechanical fixation. Advances in additive manufacturing enable functionally graded porous scaffolds and TPMS-based geometries, allowing precise control over the mechanical and biological performance of TAA implants. Surface modification strategies, including bioactive coatings, topographical texturing, and antimicrobial treatments, demonstrate potential to reduce complications such as infection and implant degradation.

Innovations in surface nanotechnology and intelligent implant systems aim to expand the applicability of TAA to broader patient populations, while emerging adaptive ‘smart’ materials promise implants that can dynamically adjust to mechanical loading and biochemical cues. These bioengineering-driven developments offer the potential to deliver more durable, individualized implants that optimize outcomes for patients with end-stage ankle arthritis. Rigorous clinical validation, long-term biocompatibility studies, and streamlined regulatory pathways will be essential for the successful translation of these technologies.

## Data Availability

No new data were created or analyzed in this study.
